# Belowground carbon allocation by trees drives seasonal patterns of extracellular enzyme activities by altering microbial community composition in a beech forest soil

**DOI:** 10.1111/j.1469-8137.2010.03321.x

**Published:** 2010-08

**Authors:** Christina Kaiser, Marianne Koranda, Barbara Kitzler, Lucia Fuchslueger, Jörg Schnecker, Peter Schweiger, Frank Rasche, Sophie Zechmeister-Boltenstern, Angela Sessitsch, Andreas Richter

**Affiliations:** 1 University of Vienna, Department of Chemical Ecology and Ecosystem ResearchAlthanstr. 14, A-1090 Vienna, Austria; 2 Department of Forest Ecology and Soils, Federal Research and Training Centre for Forests, Natural Hazards and Landscape (BFW)A-1131 Vienna, Austria; 3 AIT Austrian Institute of Technology GmbH, Bioresources UnitA-2444 Seibersdorf, Austria; 4 University of Hohenheim, Institute for Plant Production and Agroecology in the Tropics and SubtropicsD-70593 Stuttgart, Germany; 5 Institute of Soil Science, University of Natural Resources and Life SciencesPeter Jordan Straße 82, 1190 Vienna, Austria

**Keywords:** ectomycorrhizal fungi, extracellular enzyme activities, girdling, microbial community dynamics, plant–soil interactions, rhizosphere priming, seasonal dynamics, soil organic matter decomposition

## Abstract

- Plant seasonal cycles alter carbon (C) and nitrogen (N) availability for soil microbes, which may affect microbial community composition and thus feed back on microbial decomposition of soil organic material and plant N availability. The temporal dynamics of these plant–soil interactions are, however, unclear.
- Here, we experimentally manipulated the C and N availability in a beech forest through N fertilization or tree girdling and conducted a detailed analysis of the seasonal pattern of microbial community composition and decomposition processes over 2 yr.
- We found a strong relationship between microbial community composition and enzyme activities over the seasonal course. Phenoloxidase and peroxidase activities were highest during late summer, whereas cellulase and protease peaked in late autumn. Girdling, and thus loss of mycorrhiza, resulted in an increase in soil organic matter-degrading enzymes and a decrease in cellulase and protease activity.
- Temporal changes in enzyme activities suggest a switch of the main substrate for decomposition between summer (soil organic matter) and autumn (plant litter). Our results indicate that ectomycorrhizal fungi are possibly involved in autumn cellulase and protease activity. Our study shows that, through belowground C allocation, trees significantly alter soil microbial communities, which may affect seasonal patterns of decomposition processes.

## Introduction

The seasonal cycles of plants and soil microbes in temperate forests are closely linked by the availability of nutrients and labile carbon (C). Plants affect C and nitrogen (N) availability for soil microbes as a result of competition for nutrients during active growth, exudation of labile C via roots and substrate input by litterfall (Wardle *et al.*, 2004; Bardgett *et al.*, 2005; Harrison *et al.*, 2008). Microbes, in turn, control nutrient availability for plants by carrying out a wide spectrum of decomposition processes (Schimel *et al.*, 2007; Schmidt *et al.*, 2007). Thus, seasonal variations in plant growth phases as well as in microbial community composition and physiology may strongly affect C and N availability, which in turn feed back on plant and microbial processes over the course of a year.

Microbial decomposition processes in soil have been shown to be highly sensitive to the availability of labile C and N (Schimel & Weintraub, 2003). For example, the degradation of humified soil organic matter (SOM) is thought to be energy limited, leading to very slow SOM decomposition rates in soils (Kuzyakov *et al.*, 2009). The input of labile C by plant root exudation, however, may provide energy and enable microbes to degrade SOM to gain limiting nutrients (Paterson, 2009). Plant exudates may also fuel decomposition of other complex substrates, such as soil proteins (De Nobili *et al.*, 2001; Weintraub *et al.*, 2007) or litter (Subke *et al.*, 2004). Complementarily, many studies have shown that changes in N availability for soil microbes lead to alterations in decomposition rates of SOM and litter (Waldrop & Zak, 2006; Allison *et al.*, 2008; Keeler *et al.*, 2009). Together, these findings indicate that the availability of labile C and N controls the decomposition of complex substrates and thus the availability of nutrients for plants.

The mechanism by which C and N availability may control decomposition rates remains unclear. One possible explanation for this phenomenon may be the tight coupling between microbial community structure and function that has been proposed recently (Prosser *et al.*, 2007; Strickland *et al.*, 2009). As a result of the different nutrient demands and growth characteristics of specific microbial groups, altered C and N availability may favour the growth of certain microbial groups over others, thereby leading to microbial community shifts (Brant *et al.*, 2006). This may, in turn, strongly influence decomposition processes, as different microbial groups may exhibit different capacities to degrade high molecular weight substances, such as lignin, cellulose or humified SOM.

In temperate ecosystems, a strong effect of seasons on plant–soil interactions leads to seasonally variable C and N availabilities for both plants and microbes. As described above, C and N availability may be – together with abiotic factors – major drivers of microbial decomposition processes in soils. Microbial decomposition of SOM, however, represents a key driver of the forest’s C and N cycle, which highlights the need for a better understanding of how this process is influenced by plant-mediated C and N availability. Unraveling the effects of aboveground–belowground relationships on ecosystem processes and function on seasonal time-scales still remains one of the major challenges in ecosystem ecology (Bardgett *et al.*, 2005). To improve our understanding of those relationships, it is, however, necessary to gain a deeper insight into the link among C and N availability, microbial community dynamics and microbial decomposition processes in a seasonal context.

The aim of this study was to explore this link over the course of 2 yr in a temperate forest. We hypothesized that the seasonal changes in microbial community composition and physiology are driven by C and N availability in addition to abiotic factors; and that microbial community composition affects specific decomposition processes mainly through the production of distinct sets of extracellular enzymes. We tested these hypotheses by analysing the seasonal patterns of nutrient availability, microbial community structure and decomposition processes in monthly to bimonthly measurements over a period of 2 yr. Additionally, we experimentally altered the C and N availability and C:N stoichiometry through N fertilization and tree girdling. Tree girdling has been shown to effectively cut off the translocation of photoassimilates from the canopy to the roots, thereby preventing the exudation of labile C from plant roots into the soil (Högberg *et al.*, 2001; Subke *et al.*, 2004; Scott-Denton *et al.*, 2006). To link microbial community composition to decomposition processes, we investigated whether seasonal or treatment-specific microbial community changes were related to changes in specific enzyme activities.

## Materials and Methods

### Study site

Our study site was located in a mature beech forest (*Fagus sylvatica* L.) *c*. 40 km south-west of Vienna, Austria (510 m asl). The age of the trees was on average 65 yr. The soil was a dystric cambisol (over flysh) with a pH of between 4.5 and 5.1 (CaCl_2_). Organic C and total N content comprised 7.45% and 0.48% of dry soil, respectively. Despite the proximity to the city of Vienna, the site received an N input through atmospheric deposition of only 12.6 kg N ha^−1^ yr^−1^ (Kitzler *et al.*, 2006).

### Experimental set-up

The selection of control and treatment plots followed a randomized block design. First, three blocks within an area of *c*. 5400 m^2^ were selected based on a geobotanic characterization, such that each block had more or less homogenous vegetation and similar soil properties. Within each block, two control plots, two fertilization plots (5 × 5 m each) and one girdling area (20 × 20m) were chosen randomly. Two girdling plots were installed in the central 10 × 10 m of each girdling area. Each block thus contained two replicate plots of each treatment.

The fertilization treatment plots were fertilized once a month (after the soil sampling) with 29.7 g NH_4_NO_3_ dissolved in 2 l of water to give a final N fertilization rate of 50 kg ha^−1^ yr^−1^. The fertilizer was evenly distributed on each plot by spraying. The first fertilizer application was on 9 May 2006, 1 month before the first sampling. On the same day, all trees within each girdling area were girdled by ripping off a 20-cm strip of bark around the stem at a height of *c*. 1.50 m. Understory plants, which consisted mainly of beech seedlings, a few herbs and sedges, were removed from all plots (girdling, control and fertilization). Understory plants were again removed in the following spring.

### Soil sampling

Soil samples were taken from the upper 5 cm of mineral soil (A horizon). Soils from control plots were sampled every month between June 2006 and June 2008 (24 samplings). Soils from fertilized and girdled plots were sampled every 2 months, except for June and July 2007 (which were both sampled; 13 samplings in total). Four subsamples were taken from each of the six replicate plots for treatments and controls and pooled to give one replicate. Sampling was based on a predetermined sampling scheme in order to avoid sampling of already disturbed soil. Soil samples were carefully sieved (2 mm), freed from visible roots by hand-picking and kept at 4°C until further processing. All extractions were carried out on fresh soils within 4 d after sampling.

### Fine-root biomass

Fine-root biomass was determined 4, 14 and 28 months after girdling. The determination of root biomass 4 months after girdling was conducted in a parallel girdling experiment at the same study site, which started in May 2008 (three new girdling areas, with the same outline and experimental set-up as the girdling experiment described above). Thus, root biomass was determined in July 2007 (14.5 months after girdling) and September 2008 (4 and 28 months after girdling). Five soil cores (7 cm in diameter and 14.5 cm in height) were taken from the upper mineral soil (A horizon) of each of the six replicate control and girdled plots. For each soil core, fine roots (diameter < 1 mm) were carefully separated from coarse and visibly dead roots, thoroughly washed and weighed to determine fine-root biomass. Fine roots were pooled for each replicate plot. Mycorrhizal colonization was determined from aliquots of pooled samples.

### Determination of mycorrhizal root colonization

Entire root subsamples were evenly dispersed in a 9-cm-diameter Petri dish, and root length was measured using a line intersect method at ×30 magnification (Newman, 1966). The degree of mycorrhizal root colonization was determined by counting all ectomycorrhizal root tips in four randomly selected squares making up 11% of the total area of the Petri dish.

### Soil moisture and temperature

Soil moisture was detected gravimetrically in soil samples. Soil temperature was measured using Pt100 sensors (Kucera Company, Brno, Slovakia) at 5 cm depth and data were collected every 0.5 h. The soil temperature data presented are means of soil temperature measurements collected during the 6 d time period preceding the sampling date.

### Dissolved organic carbon (DOC) and total N

DOC and total dissolved N (dN) were measured in soil water extracts (2 g of fresh soil was extracted with 20 ml of analytical grade water; extracts were stored at × 20°C) using a TOC/TN analyzer (TOC-V CPH E200V/TNM-1 220V; Shimadzu, Vienna, Austria).

### Phospholipid fatty acids (PLFAs)

PLFAs were analyzed using a modified procedure described in Frostegard *et al.* (1991). Samples were processed within 1 d after sampling. Total lipids were extracted with chloroform/methanol/citric acid buffer (0.15 M), pH 4.0 (1 : 2 : 0.8, v/v/v). Neutral lipids were separated from phospholipids on silica columns (Supelco, LC-Si SPE, Vienna, Austria) by elution with chloroform, acetone and methanol. After adding methyl-nonadecanoate (19:0) as an internal standard, phospholipids were converted to fatty acid methyl esters (FAME) by alkaline methanolysis. Dried FAMEs were redissolved in isooctane and analyzed by gas chromatography (HP G1530A, Agilent, Vienna, Austria) on a DB23 column (Agilent, Vienna, Austria). A bacterial FAME mix (Supelco) was used as qualitative standard. Concentrations of FAMEs were calculated using the internal standard (19:0) peak as a reference.

We used i15:0, a15:0, i16:0, i17:0 and a17:0 as indicators for Gram-positive bacteria, 18:1ω7, cy17:0, 16:1ω7, 16:1ω9, cy18:0, cy19:0 and 16:1ω5 as indicators for Gram-negative bacteria, and the sum of Gram-positive and Gram-negative biomarkers together with 18:1ω5, 17:0, 15:0, 17:1ω6 and 17:1ω7 as a measure for total bacteria. The biomarkers 18:2ω6,9, 18:1ω9 and 18:3ω3,6,9 are frequently used as fungal markers (Hill *et al.*, 2000; Leckie, 2005; Högberg, 2006; Joergensen & Wichern, 2008). However, as they have also been found to occur in plants (Zelles, 1997; Laczko *et al.*, 2004) we measured the concentrations of these biomarkers in beech roots and calculated that the possible contribution of root-borne PLFAs (based on fine-root biomass measurements and the assumption that 95% of roots were removed by sieving) to our soil samples was ≤ 0.61% for 18:2ω6 and 18:1ω9 (or ≤ 0.31% in girdled plots) and up to 4.1% for 18:3ω3,6,9 (or 1.2% in girdled plots). Thus, the observed fine-root loss in girdled plots would account for 1.25, 0.79 and 6% of the observed decrease in 18:1ω9, 18:2ω6 and 18:3ω3,6,9, respectively (Kaiser *et al.*, 2010).

We used the sum of all PLFAs described above together with the PLFAs 20:2ω6,9 (protozoa), 10Me16:0 (actinomyceta) and 14:0, i14:0, 16:0, 19:1ω8, i17:1ω8, 18:0, 16:1ω11, 16:1ω6, 19:1ω7, 20:0, 14Me15:0 and 20:1ω9, which are not specific for any microbial group, as a measure of total microbial biomass.

### Extracellular enzymes

Potential extracellular enzyme activities were measured using microplate fluorometric and photometric assays. All activities were measured within 48 h after sampling of soils. One gram of sieved soil was suspended in 100 ml of sodium acetate buffer (100 mM, pH 5.5) and ultrasonicated at low energy (Stemmer *et al.*, 1998; Marx *et al.*, 2001). β-1,4-Cellobiosidase (‘cellobiosidase’), β-1,4-N-acetylglucosaminidase, chitinase/lysozyme (‘chitinase’) and leucine amino-peptidase (‘peptidase’) were measured fluorimetrically (Marx *et al.*, 2001; Saiya-Cork *et al.*, 2002). Two hundred microliters of soil suspension and 50 μl of substrate (4-methylumbelliferyl-β-d-cellobioside, 4-methylumbelliferyl-N-acetyl-β-d-glucosaminide, 4-methylumbelliferyl-β-d-N,N′,N′′-triacetylchitotrioside and l-leucine-7-amido-4-methyl coumarin, respectively) were pipetted into black microtiter plates in three analytical replicates. Methylumbelliferyl (MUF) was used for calibration of cellobiosidase, N-acetylglucosaminidase and chitinase, whereas aminomethylcoumarin (AMC) was used for calibration of leucine amino-peptidase. Plates were incubated for 140 min in the dark and fluorescence was measured at 450 nm emission at an excitation at 365 nm (using a Tecan Infinite M200 fluorimeter, Werfen, Austria).

Different enzymes with different abilities to cope with steric hindrance may be involved in the degradation of polymers, such as chitin. We therefore assayed chitinases with two types of substrate: 4-methylumbelliferyl-β-d-N,N′,N′′-triacetylchitotrioside, consisting of three units of N-acetyl-β-d-glucosaminide (component of chitin), and 4-methylumbelliferyl-N-acetyl-β-d-glucosaminide, consisting of only one unit.

Phenoloxidase and peroxidase activities were measured photometrically according to standard methods (Sinsabaugh *et al.*, 1999), with small modifications. Subsamples were taken from the soil suspension (see above) and mixed with a 20 mM l-3,4-dihydroxyphenylalanin (L-DOPA, Sigma-Aldrich, Vienna, Austria) solution (1 : 1). Samples were shaken for 10 min and centrifuged, and aliquots were pipetted into microtiter plates (six analytical replicates per sample). Half of the wells additionally received 10 μl of a 0.3% H_2_O_2_ solution for measurement of peroxidase. Absorption was measured at 450 nm at the starting time-point and after 20 h. Enzyme activity was calculated from the difference in absorption between the two time-points divided by the molar extinction coefficient, which had been determined in a preliminary experiment.

### Actual extracellular enzyme activities

‘Actual’ enzyme activities were measured without substrate and buffer additions, but with the addition of toluene to inhibit microbial uptake of enzymatic products, leading to their accumulation in the soil suspension (Boschker *et al.*, 1995; Watanabe & Hayano, 1995; Lipson *et al.*, 1999; Weintraub & Schimel, 2005). This method has been used before to determine actual protease activities (accumulation of amino acids; Lipson *et al.*, 1999; Weintraub & Schimel, 2005) and to determine polysaccharide degradation (accumulation of glucose and xylose; Boschker *et al.*, 1995). We measured both activities in the same assay: 8 g of fresh soil was mixed with 80 ml of distilled water and 0.8 ml of toluene in a glass jar and shaken for 4 h. Subsamples for the detection of amino acids and glucose were sampled after 0.5 and 4 h. For measurement of amino acid production, subsamples were centrifuged and supernatants were mixed with the same volume of a solution containing 0.11 M trichloroacetic acid, 0.22 M sodium acetate and 0.33 M acetic acid, to stop further enzyme activity (Weintraub & Schimel, 2005). Samples were analyzed for amino acids using a photometric assay with ninhydrin (Moore, 1968). For measurement of glucose production, subsamples were centrifuged twice at 15 000 ***g*** to remove soil particles and microbes and were incubated at 100°C for 15 min to stop enzyme activities. Samples were then analyzed for glucose by HPLC (ICS-3000; CarboPac PA20 column; 10 mM NaOH, with pulsed amperiometric detection, DIONEX, Vienna, Austria). In a preliminary experiment, the accumulation of both amino acids and glucose was shown to be linear up to 6 h. Glucose accumulation in the soil solution was probably caused by cellulase and/or amylase activity (Boschker *et al.*, 1995). We detected some accumulation of cellobiose and maltose in the soil solution, but concentrations were much lower than that of glucose, indicating that these intermediate products were rapidly further degraded. Thus, we used glucose production as a proxy for the combined action of cellulose and amylase. We ascribed accumulation of amino acids in the soil solution to protease activity (Weintraub & Schimel, 2005).

### Statistics

We carried out a canonical correspondence analysis (CCA; Anderson & Willis, 2003; Gonzalez *et al.*, 2008) on a data set of 11 samplings (bimonthly from August 2006 to May 2008; August 2007 had to be excluded because of missing data for phenoloxidase and peroxidase activity (*n*= 195)). A total of 37 individual PLFA biomarkers were used as the community matrix (nmol PLFAs g^−1^ dry soil), whereas different sets of constraining variables (abiotic conditions, C and N pools and microbial processes) were used to compare the influence of different groups of environmental parameters on microbial community composition. The contribution of constrained variability to total community variability (= fraction of variability explained by the parameter) in each analysis was used as a measure of the influence of the respective parameter set on community variation (used in Table 2). Results from a CCA conducted with all parameters (except glucose production, which was not available for the first two samplings) as the constraining matrix are presented graphically in more detail (see Fig. 6 below). We conducted a linear regression analysis (ordinary least square regression) between all individual PLFA biomarkers and all measured soil parameters for the 2-yr sampling period. All statistical analyses were performed in R 2.8.1 (http://www.r-project.org/, R-package *vegan*).

## Results

### Climate and abiotic conditions

**Fig. 1:**
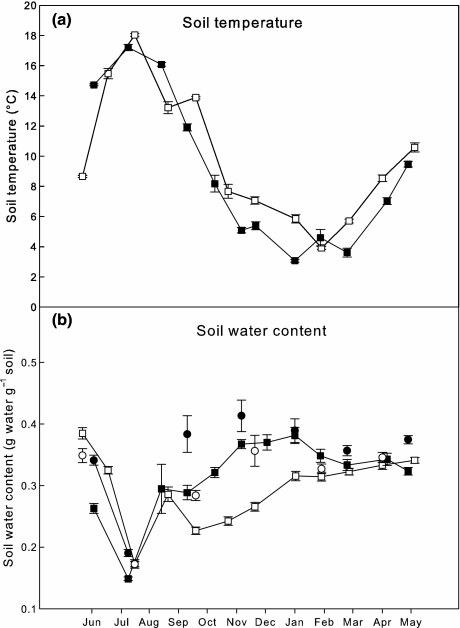
(a) Mean soil temperature (at 5 cm depth) during the two sampling years (2006/2007 and 2007/2008). Each data point represents the average of the daily mean soil temperatures on the 7 d preceeding each sampling date (for all treatments: open symbols, year 1; closed symbols, year 2). (b) Gravimetric soil water content (0–5 cm depth) for control and girdled plots (squares, controls; circles, girdled plots) during the two sampling years. Error bars indicate 1 SE (*n* = 6).

Temperature and precipitation patterns were different between the two sampling years (June 2006 to May 2007 and June 2007 to May 2008). The first autumn and winter period (September 2006 to January 2007) was on average 2.2°C warmer than the second autumn/winter, and exceptionally dry, with almost no precipitation and no snow cover. By contrast, the second sampling year was characterized by continuous snow cover from November to March. Soil water content ranged from 25 to 30% between October and January of the first sampling year, and from 30 to 40% in the same period of the second year (Fig. 1b). In both years, soil water content was lowest in August (23%). Soil temperature was *c*. 1.3°C higher between September and May of the first year compared with the second year (Fig. 1a).

Girdling affected soil water content significantly. The magnitude of this effect, however, depended on season. Soil moisture was significantly higher in girdled plots from September to December of both years (by 5–10%), and in spring and summer of the second year. No difference, however, was observed between January and March of both years (Table 1, Fig. 1b). Fertilization did not affect soil water content. There was no difference in soil temperature between treatments and controls.

**Table 1:** Significance of the effects of girdling, fertilization and sampling month on various soil parameters derived from an analysis of variance (ANOVA)

	Year 1						Year 2					
	Girdling			Fertilization			Girdling			Fertilization		
Soil parameter	m (df = 5)	g (df = 1)	g × m (df = 5)	m (df = 5)	f (df = 1)	f × m (df = 5)	m (df = 6)	g (df = 1)	g × m (df = 6)	m (df = 6)	f (df = 1)	f × m (df = 6)
Soil water content	***	***	***	***	ns	ns	***	***	*	***	ns	ns
Cellobiosidase	***	ns	ns	***	ns	ns	***	ns	°	***	ns	ns
Chitinase	***	°	ns	***	ns	ns	***	**	ns	**	ns	ns
N-Acetylglucosaminidase	***	ns	ns	***	ns	ns	***	ns	**	***	ns	ns
Leu-peptidase	***	ns	ns	***	ns	ns	***	ns	***	***	ns	ns
Phenoloxidase^a^	***	°	***	***	ns	ns	***	ns	***	***	*	***
Peroxidase^a^	***	***	**	***	ns	ns	***	ns	***	***	***	***
Actual protease^b^	***	***	ns	**	ns	ns	***	***	°	***	ns	*
Glucose production^c^	***	***	**	***	ns	ns	***	**	***	***	°	ns
DOC	**	ns	ns	**	ns	ns	***	ns	ns	***	ns	*
Total dissolved N (dN)	***	***	***	***	*	ns	***	***	***	***	**	ns
DOC: dN	***	***	***	***	***	**	***	***	***	***	**	**
CCA1	***	ns	ns	***	*	***	***	ns	ns	***	ns	°
CCA2	*	***	**	ns	ns	ns	**	***	ns	*	*	ns

Presented are levels of significance resulting from factorial ANOVAs (with month and treatment as factors) conducted separately for the girdling and fertilization treatments, and for each year of the experiment. DOC, dissolved organic carbon; m, month of sampling; g, girdling; f, fertilization; g × m and f × m, interactions of month and treatment. The number of replicates for the first and second years was *n* = 72 (six samplings) and *n* = 84 (seven samplings), respectively. Degrees of freedom (df) for each factor are indicated in the table headings, with the following exceptions.

a *n* = 72 and df (m and m × treatment) = 5 for year 2;

b *n* = 60 and df (m and m × treatment) = 4 for year 1;

c *n* = 48 and df (m and m × treatment) = 3 for year 1. CCA1, CCA2, sampling scores of the first two axes of the canonical correspondence analysis (Fig. 6, Supporting Information Fig. S2).

Significance levels:

*** *P* < 0.001;

** *P* < 0.01;

* *P* < 0.05;

° *P* < 0.1.

ns, not significant.

### Effect of girdling on tree vitality

In the first year, leaf senescence started *c*. 3 wk earlier in girdled trees. In the following spring, however, girdled trees still exhibited a full flush of leaves. The amount of leaf litter in the second sampling year was similar in all plots, although litter fall occurred earlier in girdled plots (34% of total leaf litter fall in girdled plots but only 13% in control plots took place before the end of September 2007; Supporting Information Fig. S1). In spring 2008 (2 yr after girdling), 40% of girdled trees did not produce new leaves.

### Fine-root biomass and ectomycorrhizal colonization

**Fig. 2:**
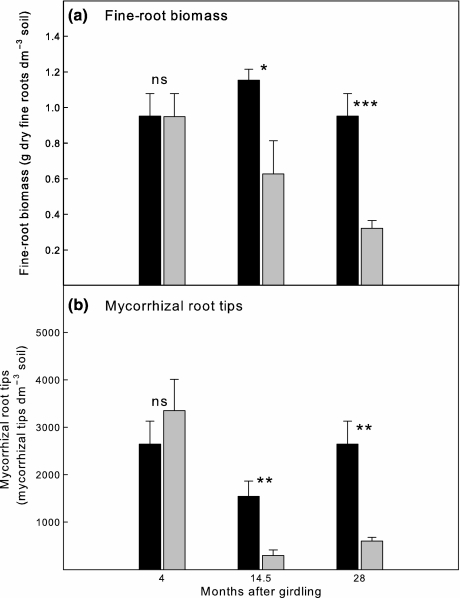
Dry fine-root biomass (a) and ectomycorrhizal root tips (b) per dm^−3^ of soil in control and girdled plots at 4, 14.5 and 28 months after girdling. Black bars, controls; gray bars, girdled plots. Error bars indicate 1 SE (*n* = 6). Asterisks indicate statistical significant differences between control and girdled plots for each time-point (ns, not significant; *, *P*< 0.05; **, *P*< 0.01;***, *P*< 0.001).

Fine-root biomass in control plots was *c*. 1–1.2 g dry fine roots dm^−3^ soil in the upper 14.5 cm of soil. We observed no difference in fine-root biomass or ectomycorrhizal root tip colonization between control and girdled plots 4 months after girdling (Fig. 2). Fine-root biomass decreased by 45 and 60% in girdled plots 14 and 28 months after girdling, respectively. Mycorrhizal root colonization was reduced by 60% 14 months after girdling, but only by 27% 28 months after girdling. However, because of the smaller amount of vital roots at the latter time-point, the total amount of mycorrhizal root tips per cm^3^ soil was decreased by *c*. 70% for both 14 and 28 months after girdling (Fig. 2).

### DOC and N levels

**Fig. 3:**
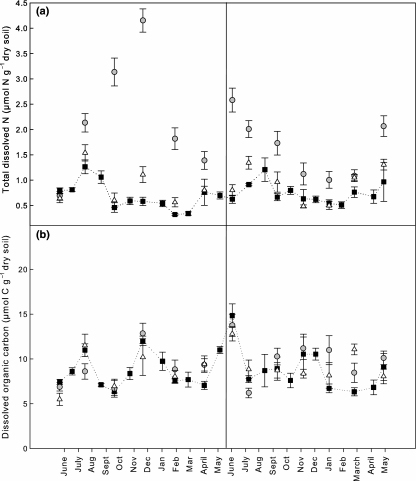
Total dissolved nitrogen (a) and dissolved organic carbon (b) in soil water extracts over the course of the two sampling years (squares, controls; circles, girdled plots; triangles, fertilized plots; dotted lines connect control values to show the seasonal trend). All data are plotted on the actual sampling date; ticks are for the 15th day of each month. Error bars indicate 1 SE (*n* = 6).

In both years, DOC content in soil water extracts was low in early autumn (September to October), increased in late autumn (November to December) and decreased again to low values between January and April, followed by a sharp increase in May (Fig. 3b). Thus, autumn and winter dynamics were similar between the two sampling years, probably indicating a general seasonal trend. By contrast, we could not observe similar DOC dynamics in early and late summer of the two years. Tree girdling decreased DOC in soil water extracts predominantly in July of both years (−21.7% (*P*= 0.058) and −20% (*P*= 0.044), respectively), but did not affect DOC during autumn and winter (Fig. 3b). Total (= organic and inorganic) dissolved N in soil water extracts peaked around mid-summer (August) in both years, followed by a sharp decrease in autumn to low levels in winter (Fig. 3a). Levels of dissolved N were significantly enhanced by fertilization and girdling, on average (over the whole sampling period) by 28 and 170%, respectively (*P*< 0.001 and *P*< 0.05, respectively; Fig. 3, Table 1).

### Soil enzyme activities

**Fig. 4:**
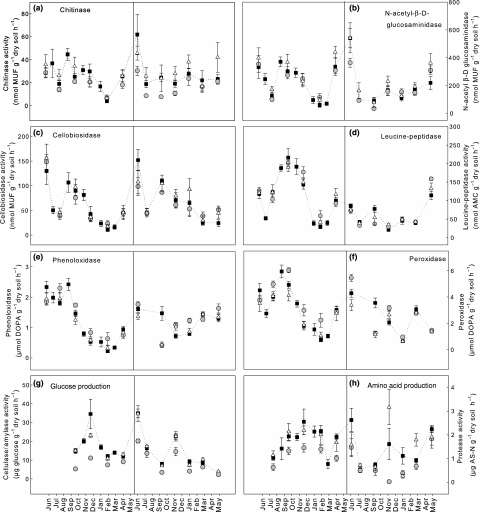
Soil extracellular enzyme activities in control, fertilized and girdled plots throughout the 2-yr sampling period (squares, controls; circles, girdled plots; triangles, fertilized plots; dotted lines connect control values to show the seasonal trend). (a–f) Potential activities (activities measured using fluorometric and photometric methods after addition of a specific substrate). (g, h) Actual enzyme activities (no substrate added). All data are plotted on the actual sampling date; ticks are for the 15th day of each month. MUF, Methylumbelliferyl; AMC, Aminomethylcoumarin; DOPA, L-3,4-dihydroxyphenylalanin; AS-N, Amino acid-nitrogen. Error bars indicate 1 SE (*n* = 6).

Soil enzyme activities were high in spring, late summer and autumn, and low in mid-summer and winter (Fig. 4). The spring peak was highly synchronous for all measured enzymes (in 2007), starting with fairly low levels in March and increasing to maximum levels in June, followed again by a summer decline. The second (‘autumn’) peak occurred at different times for different enzymes: (potential) phenoloxidase and peroxidase showed maximum activities already in late summer (around September), whereas glucose production (by actual cellulase/amylase activity; Fig. 4g) and actual protease activity (Fig. 4h) had their autumn maximum around November. At this time, phenoloxidase and peroxidase activities had already dropped to very low levels (Fig. 4e,f).

We found no significant effect of girdling or fertilization on enzymes measured using fluorescent substrates (chitinase, cellobiosidase, N-acetylglucosaminidase and peptidase) during the first year (Table 1, Fig. 4a–d). In the second year, chitinase was found to be significantly decreased in girdled plots (Table 1, Fig. 4a).

‘Actual’ protease and cellulase activities were markedly reduced in girdled plots, whereas phenoloxidase and peroxidase activities were significantly enhanced (Fig. 4e–h, Table 1). These shifts in activities occurred already in the first summer after girdling. Fertilization affected phenoloxidase and peroxidase activity only in the second year (Table 1, Fig. 4e,f).

### Microbial biomass

**Fig. 5:**
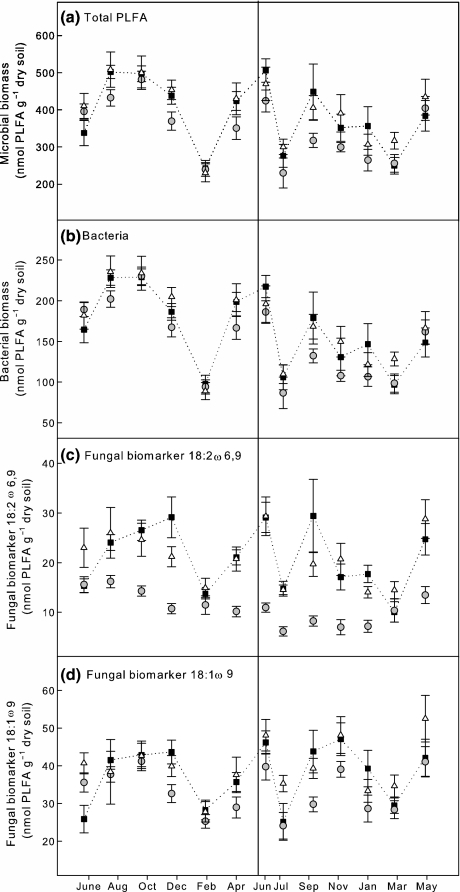
Total phospholipid fatty acid (PLFA; a), bacterial (b) and fungal (c, d) biomarkers in control, girdled and fertilized plots over a period of 2 yr (squares, controls; circles, girdled plots; triangles, fertilized plots; dotted lines connect control values to show the seasonal trend). All data are plotted on the actual sampling date; ticks are for the 15th day of each month. Error bars indicate 1 SE (*n* = 6).

The annual course of the amount of total PLFAs, as a measure of microbial biomass, was similar in the two sampling years in control plots, apart from a small time shift (Fig. 5a). Microbial biomass showed two phases of lower values during the course of a year: the first in June (July in the second year) and the second in February (March in the second year). In both years we observed a sharp increase of biomass in spring and a period of high values in autumn (Fig. 5a, Table S1).

Girdling decreased the total amount of PLFAs by 15% on average between July 2006 and January 2008 (2–20 months after girdling). In the same time period, the sum of bacterial biomarkers was decreased by *c*. 14%, the amount of the fungal biomarker 18:1ω9 by 16% and the fungal biomarker 18:2ω6,9 by as much as 51% (Fig. 5).

Fungi are thought to contain smaller amounts of PLFAs per g biomass compared with bacteria because of a higher ratio of cell volume to cell surface, which makes it difficult to estimate the real fungi to bacteria ratio from PLFA data (Joergensen & Wichern, 2008). Thus, the reduction of 51% of the 18:2ω6,9 biomarker may translate into a much greater reduction of the total microbial biomass than the 15% loss of total PLFAs suggests. It is worth noting that in girdled plots the seasonal trend was maintained for bacteria and the fungal biomarker 18:1ω9, but not for the fungal biomarker 18:2ω6,9, which had already started to decrease strongly 2 months after girdling (Fig. 5). Fertilization had no significant effect on the total amount of bacterial or fungal PLFA biomarkers.

### Microbial community composition

We conducted a canonical correspondence analysis (CCA) in order to assess how far the variation observed in the microbial community composition was related to specific environmental parameters. By contrast to unconstrained multivariate analysis, CCA displays only the part of the variation in the community data that can be explained by the used constraints. Our analysis revealed that soil temperature and soil water content were related to 20% of the PLFA variability, whereas labile C and N (DOC and dissolved N) could only explain 4.3% of the total community variability (Table 2). Enzyme activities, however, were linked to 24% of the variability in the community matrix. Of all enzymes, phenoloxidase, peroxidase and peptidase were most strongly related to community structure (19.9%), whereas other enzymes explained much smaller parts of the variance (e.g. N-actyl-glucosaminidase, chitinase and cellobiase only 5.4%; Table 2).

**Table 2:** Relationship between different soil parameters and soil microbial community composition

Set of constraints	Explained fraction of variability (%)
Soil temperature and soil water content	20.3
DOC and dN	4.3
N-acetyl-β-d-glucosaminidase, chitinase and cellobiosidase	5.4
Peptidase	12.1
Phenoloxidase and peroxidase	15.9
Phenoloxidase, peroxidase and peptidase	19.9
Actual protease and cellulase/amylase*	6.3
All enzymes	24.2
All parameters	36.0

Presented are the contributions of constrained variability to total community variability by canonical correspondence analysis (CCA) conducted with different sets of constraining variables. CCA was based on data from bimonthly samplings of control, fertilized and girdled plots between August 2006 and May 2008 (with the exclusion of August 2007 because of missing peroxidase and phenoloxidase data; 11 samplings; *n* = 195). Additionally, data for July 2006 are missing for cellulose/amylase actitivity

* (10 samplings; *n* = 177). DOC, dissolved organic carbon; dN, total dissolved nitrogen.

**Fig. 6:**
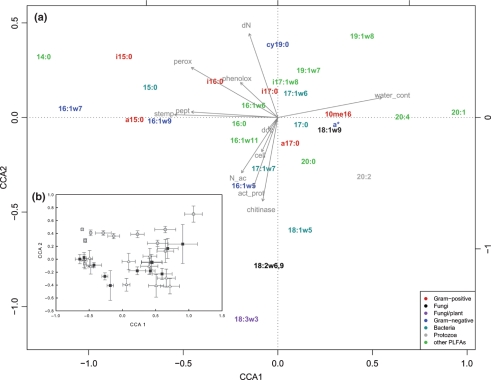
Relationship between microbial community composition and various soil parameters analyzed by canonical correspondence analysis (CCA). Phospholipid fatty acid (PLFA) data were used as the community matrix; environmental and microbial process data as a constraining matrix. The analysis was based on data from all samplings between 6 August and 8 May, except for 7 July. (a) The distribution of individual PLFA biomarkers (weighted scores) is shown on the first two axes. Additionally, the biplot scores of constraining variables depicting the influence of soil parameters are shown on these axes. Biomarkers are colored according to their allocation to specific microbial groups. a*, 18:1ω7 and cy17:0; N_ac, N-acetyl-β-D-glucosaminidase; cell, cellobiosidase; stemp, soil temperature; act_prot, actual protease; pept, leucine-peptidase. (b) The distribution of the bimonthly samplings for the two sampling years is shown along the first two axes. Circles, girdled plots; squares, control plots; triangles, fertilized plots. The contribution of constrained variability to total variability was 36%, of which CCA1 accounts for 74.8% and CCA2 for 12.5%. CCA1 and CCA2 were both significant (*P*< 0.001; permutation test). The distribution of bimonthly samplings in the course of time is shown in Supporting Information Fig. S2.

Altogether, *c*. 36% of the variability of the microbial community matrix throughout seasons and treatments could be related to a combination of enzymatic activities and environmental parameters (Fig. 6, Table 2). Of the constrained variability, CCA axis 1 (CCA1) accounted for 72.2%, and CCA axis 2 (CCA2) for 12.4%. Both CCA1 and CCA2 were significant (*P*< 0.001; permutation test). We found significant effects of seasons and girdling on microbial community composition along these two axes (Fig. 6, Table 1). Different sampling months were mainly separated on CCA1 whereas control and girdled plots could be separated on CCA2 (Fig. 6b). No general trend for specific microbial groups (Gram-negative bacteria, Gram-positive bacteria, all bacteria, fungi) was observed along CCA1, indicating that seasonal microbial community shifts were caused by changes within all groups. The community shift along CCA2 (reflecting the effect of girdling) was dominated by the influence of two fungal biomarkers (18:2ω6,9 and 18:3ω3,6,9) and one bacterial biomarker (18:1ω5), which were negatively correlated to that axis (indicating reduced abundance of these markers in samples from the girdling treatment). The influence of the third fungal biomarker (18:1ω9) was comparably low (Fig. 6).

Of all sampling months, February of the first year and November of the second year had the highest scores on CCA1 (Supporting information Fig. S2). Both were the first months in which winter conditions (high soil moisture and low soil temperature) occurred in the respective year. Spring and summer months were characterized by lower scores (Fig. S2). Soil temperature and soil water content as well as peroxidase and peptidase activities exhibited a strong effect on CCA1, indicating that they were closely related to seasonal variation of microbial community composition (Fig. 6).

A regression analysis of each individual biomarker with each microbial process revealed interesting links between microbial processes and community structure (Table 3). The results of this regression analysis showed that enzymatic activities can be separated into three groups, each of them relating to a specific set of individual PLFA biomarkers. First, phenoloxidase and peptidase activities were correlated with peroxidase activity and all three enzymes were correlated to the same same set of individual PLFAs, including the majority of Gram-positive biomarkers (i15:0, a15:0, i16:0), certain Gram-negative biomarkers (16:1ω7, 16:1ω9) and a few general bacterial biomarkers (14:0, 16:1ω11) (Table 3). Secondly, cellobiosidase, N-acetylglucosaminidase and chitinase were correlated with each other. Overall, these enzymes did not correlate with any specific PLFA biomarker, with the exeption of N-acetylglucosa-minidase and chitinase which were correlated to the microbial biomass (sum of all PLFAs). The third group of enzyme activities, comprising ‘actual’ protease and cellulase/amylase activities, were not correlated to any bacterial biomarker, but – together with chitinase activity – to the fungal biomarkers 18:2ω6,9 and 18:3ω3,6,9 (Table 3). There was no correlation of one of the fungal biomarkers with peroxidase or phenoloxidase activity. However, when the regression analysis only for girdled plots was examined, a strong correlation (*r*^2^ = 0.54, *P*< 0.005) between 18:2ω6,9 and phenoloxidase activity was found (Table S2).

**Table 3:** Linear regressions of phospholipid fatty acid biomarkers and various soil parameters

PLFA biomarkers\Soil parameter		Water content	Soil temp	Cellob.	Chitin.	N-acet.	L-pept.	Ph.ox.	Perox.	Act. prot.	Gluc. p.	dN	DOC: dN
Gram-positive	i15:0		0.36				**0.52**	0.37	**0.56**				
	a15:0		0.30				0.50	0.33	0.47				
	i16:0		0.38				0.36	0.38	0.45				
	i17:0		0.36					0.27	0.29				
	a17:0		0.27										
Actinomycetes	10me16												
Gram-negative	18:1ω7												
	cy17:0												
	16:1ω7					0.27	0.40	0.30	**0.54**				
	16:1ω9		0.36				0.49	0.27	0.36				
	cy18:0						0.32	0.34					
	cy19:0												
	16:1ω5			0.28	0.36		0.35	0.31	0.29	0.28			
Bacteria	18:1ω5												0.27
	17:00												
	15:00		0.36				0.41	0.35	**0.55**				
	17:1ω6												
Fungi	18:2ω6				0.29					0.36	0.27		0.35
	18:1ω9												
F&P	18:3ω3				0.26					0.27	**0.57**		0.38
General	14:00		0.36				**0.55**	0.38	**0.58**				
	16:00		0.33				0.32	0.30	0.36				
	16:1ω11			0.28	0.32		0.41	0.31	0.33				
	16:1ω6		0.37		0.27		0.35		0.35				
	17:1ω6						0.25						
	18:00		0.26						0.28		0.28		
Groups	Gram+		0.36				0.47	0.36	0.50				
	Gram−												
	Bacteria		0.27				0.41	0.34	0.38				
	Fungi												
	All		0.26		0.35	0.30	0.37		0.34				
Processes	Cellob.			**1.00**	0.42	0.32							
	Chitin.			0.42	**1.00**	**0.62**					0.35		
	N-acet.			0.32	**0.62**	**1.00**				0.37			
	L-pept.						**1.00**		0.30				
	Ph.ox.		0.34					**1.00**	**0.52**				
	Perox.		0.47				0.30	**0.52**	**1.00**				
	Act. prot.					0.37				**1.00**			0.32
	Gluc. p.				0.35						**1.00**		

Presented are goodness of fit (*R*^2^) of statistical significant (*P*< 0.01) relationships between parameters; empty fields indicate that the regression was not significant. Bold values show *R*^2^ > 0.5. Regressions are based on data from control and girdling plots from all samplings over the 2-yr sampling period (mean values of each sampling). Cellob., cellobiosidase; Chitin., chitinase; N-acet., N-acetyl-glucosaminidase; L-pept., leucine-peptidase; Ph.ox., phenoloxidase; Perox., peroxidase; Act. prot., actual protease; Gluc. p., glucose production; dN, total dissolved nitrogen; F&P, fungi and plants; DOC, dissolved organic carbon; PFLA, phospholipid fatty acid.

## Discussion

Tree roots provide an important source of easily assimilable C for soil microbes and allow the establishment of a specific microbial community (Brant *et al.*, 2006). In temperate forests, an important part of this community comprises ectomycorrhizal fungi (Read & Perez-Moreno, 2003; Jones *et al.*, 2004), but other microbial groups also benefit from this source of C (Butler *et al.*, 2003). Eliminating belowground C allocation by tree girdling in our study had a twofold effect on C and N availability for microbes. First, it led to decreased DOC contents in the soil during the summer. This effect was similar to that of other girdling studies which also found small season-dependent effects of girdling on DOC contents in soil water extracts (Weintraub *et al.*, 2007; Zeller *et al.*, 2008). As DOC is a labile pool with fast turnover times, its absolute amount may provide little information about substrate supply to microbes. However, we also observed a reduced microbial biomass and a strong decline of soil CO_2_ efflux (B. Kitzler *et al*., unpublished), confirming that the availability of easily assimilable C was substantially depleted in the girdling treatments. By contrast, N concentrations were strongly enhanced in the soil water of girdled plots, presumably as a result of reduced plant N uptake caused by reductions in mycorrhizal hyphae and fine roots in the soil (Jordan *et al.*, 1998). Increased dissolved inorganic N concentrations in response to girdling have also been found in other studies (Weintraub *et al.*, 2007; Zeller *et al.*, 2008; Dannenmann *et al.*, 2009) and may affect microbial community composition in addition to the loss of root C input.

The effect of girdling on soil C and N availability led to substantial changes in microbial biomass and community composition. Although only a small proportion of the bacterial community (15%) was affected, the fungal biomarker 18:2ω6,9 was reduced by 51%. This is consistent with other girdling studies, which showed a strong decline of the fungal biomarker 18:2ω6,9, but almost no response of 18:1ω9. Compared with other types of fungi, mycorrhizal fungi depend to a much higher degree on belowground C allocation by trees. It is therefore safe to assume that the reduction of fungal biomarkers after girdling was related mainly to the reduction of mycorrhizal fungi, as has also been suggested by others (Högberg, 2006; Högberg *et al.*, 2007; Yarwood *et al.*, 2009). Surprisingly, we found a strong decrease of 18:2ω6,9 just 2 months after girdling (Fig. 5), although we did not observe any decrease of mycorrhizal root colonization 4 months after girdling (Fig. 2). The latter is consistent with the results of another beech girdling experiment (Dannenmann *et al.*, 2009), in which girdling had no effect on mycorrhizal root colonization in the first year. In our study, however, mycorrhizal root colonization decreased by as much as 60% 14 months after girdling (Fig. 2). Our results suggest that reducing the input of labile root C initially resulted in a strong decrease of the mycorrhizal extramatrical hyphal network in the soil within the first 2 months, followed only later by a decrease in ectomycorrizal root tips and a loss of fine-root biomass.

Potential activities of cellobiosidase, N-acetylglucosaminidase and peptidase were not significantly affected by girdling, demonstrating that they were not linked to root exudations. These enzymes (measured using relatively simple low-molecular-weight substrates) possibly represent a general trait of microbial groups other than mycorrhizal fungi and rhizosphere bacteria, which were not affected by girdling. In contrast to the above-mentioned enzyme activities, our results showed a clear decrease of actual cellulase and protease activities in girdled plots already in the first year, especially at times when these rates were at peak values in control plots; that is, in spring and autumn (Fig. 4). The diminishing of the spring peak in girdled plots indicates that this peak is linked to root exudates. Spring exudates may have accelerated the turnover of microbes, leading to a flush of proteins and carbohydrates (De Nobili *et al.*, 2001; Kuzyakov, 2002; Phillips *et al.*, 2008). The autumn peak in enzyme activity, in contrast, may be explained by the input of fresh litter at that time. Approximately 70% of N-rich low-order roots are thought to have turnover times of < 1 yr (Kielland *et al.*, 2006; Guo *et al.*, 2008; Pritchard & Strand, 2008) and a large proportion of fine-root mortality is usually found at the end of the growing season (Ruess *et al.*, 2003). Together with leachates from leaf litter, this peak of fine-root mortality in autumn may therefore have led to a large input of cellulose and protein into the soil and subsequently to increased glucose and amino acid production.

As there was no reduction in overall fine-root biomass in the first year of girdling, we assume that this input of fresh substrates did occur in control as well as in girdled plots. Nevertheless, girdling strongly reduced this autumn peak of glucose and amino acid production (by > 50%) in November of both years (Fig. 4) This reduction was correlated to a strong reduction of the autumn and winter peak of fungal biomarkers in girdled plots (Fig. 5b, Table 3), suggesting that mycorrhizal fungi may be closely involved in protease and cellulase/amylase activity in autumn. At the time when amino acid and glucose production rates exhibited their autumn peak (in November), (potential) phenoloxidase and peroxidase activities strongly decreased to very low values (Fig. 4). Thus, we observed a temporal shift in enzyme activities from an early autumn maximum of oxidative enzymes to a late autumn maximum of hydrolytic enzymes. While the maxima of cellulase and protease activities can be readily explained by increased substrate inputs in late autumn (see the Discussion section), the reasons for the drop in oxidative enzymes by *c*. 70% from September to December are less clear. Our data suggest that it is not likely that this drop was driven by abiotic factors. For example, October 2006 exhibited warmer soil temperatures than September, although there was already a strong decrease (by 45%) in oxidative enzymes in this period (Fig. 4). Potential measurements of oxidative enzyme activities may, however, reflect the abundance of the microbes producing them, rather than the actual rates of these enzyme activities. Phenoloxidase and peroxidase catalyze the key reactions in the degradation of lignin and humified SOM and have to be carried out by specialist microbes (Hatakka, 2001; Baldrian, 2009). However, specialists for SOM degradation will only have an advantage over others when other sources of nutrients (such as fresh organic matter (FOM)) are scarce (Fontaine *et al.*, 2003; Kuzyakov *et al.*, 2009; Paterson, 2009), which is possibly the case during summer. Plant root exudates may provide a constant energy supply at low N levels during summer, thereby creating optimal conditions for SOM degraders (Fontaine *et al.*, 2003). This situation may change fundamentally when N-rich substrates, such as dying fine roots and leaf litter leachates, appear in autumn. SOM-degrading microbes may then lose their competitive advantage over microbes decomposing FOM, explaining the rapid autumn and winter decrease of phenoloxidase/peroxidase.

Competition between microbial groups could also have been responsible for the shift of enzyme activities from hydrolytic enzymes to oxidative enzymes in girdled plots. Mycorrhizal fungi are known to dominate the rooted soil layers as a result of a competitive advantage gained through access to root C, whereas saprotrophic fungi are thought to be more competitive in the litter layer (Hobbie & Horton, 2007; Lindahl *et al.*, 2007). Although ectomycorrhizal fungi have been found to be able to produce cellulases, proteases and phenoloxidases (Luis *et al.*, 2005; Cullings & Courty, 2009), saprotrophic fungi are thought to possess a much greater capacity to produce peroxidases (Luis *et al.*, 2005; Baldrian, 2009; Cullings & Courty, 2009). Girdling greatly decreased the abundance of mycorrhizal fungi, thereby possibly also giving saprotrophic fungi a competitive advantage in the rooting zone. At the same time, girdling reduced easily assimilable C in soils, which may have led to starvation of microbes, while N availability increased to very high levels (Fig. 3). According to the current understanding of rhizosphere priming, this would not favour SOM degradation, because under such conditions there is no need to mineralize N and SOM degradation to gain C would be too energy demanding (Kuzyakov *et al.*, 2009; Paterson, 2009). Nevertheless, potential oxidative enzyme activities rose in girdling plots in our experiment, suggesting that, at least in the longer term, the reduced abundance of mycorrhizal fungi may have relieved the competitive disadvantage of slow-growing specialist decomposers (e.g. saprotrophic fungi) which possess the ability to degrade SOM by releasing peroxidases and phenoloxidases (Hobbie & Horton, 2007; Baldrian, 2009).

Alternatively, reducing C input from the host tree could have led to a shift within the ectomycorrhizal community towards species with a greater ability to produce SOM-degrading enzymes or to a physiological switch of ectomycorrhizal fungi towards increased saprotrophic activity (Buee *et al.*, 2007; Talbot *et al.*, 2008; Cullings & Courty, 2009). Different mechanisms have recently been suggested which may lead to the decomposition of SOM by mycorrhizal fungi; for example, the need for an alternative C source when supplies of photosynthetates from plants are low, or, by contrast, ‘priming’ with high amounts of photosynthetates, which may accelerate the growth and activity of ectomycorrhizal fungi (Courty *et al.*, 2007; Cullings *et al.*, 2008; Talbot *et al.*, 2008). Our results suggest that, if mycorrhizal fungi were able to switch to saprotrophy at low C supply (as indicated by increased phenoloxidase and peroxidase activities in girdled plots), it was apparently not very effective, as fungal biomass strongly decreased in girdled plots. Instead, it seems plausible that fungi were primed by tree C in control plots, in which they grew to high abundances from summer to autumn and were linked to high activities of cellulase and protease, both of which diminished in girdled plots. An increase of phenoloxidase and peroxidase activities after girdling was also found by Weintraub *et al.* (2007), who suggested that this may have been caused by increasing availability of dead fine-root biomass as a result of girdling. However, because of the rapid increase of oxidative enzymes in girdled plots just 2 months after treatment and the lack of a decrease in fine-root biomass in the first 4 months (Fig. 2), we rule out this explanation for our experiment.

Regression analysis of PLFAs and enzyme activities over all seasons revealed three ‘groups’ of enzymes, each related to a specific set of individual PLFA biomarkers (Table 3). Of all measured enzyme activities, the activities of phenoloxidase, peroxidase and leucine-peptidase were those linked to the largest part of the variation (20%) in the microbial community composition, whereas cellobiosidase, N-acetylglucosaminidase and chitinase showed the weakest relation to community composition (Tables 2, 3). The high number of significant correlations between PLFA biomarkers and enzymes suggests that the observed enzyme pattern was determined to a large extent by microbial community dynamics, rather than by individual physiological response of microbes to varying nutrient and energy availability. Correlations between phenoloxidase, peroxidase and individual PLFAs were stronger and also involved the fungal biomarker 18:2ω6,9 (*R*^2^ = 0.54, *P* = 0.007; Table S2) when girdled plots were analyzed alone, indicating that more microbial groups were connected to these enzyme activities after girdling (Table S2).

Tree C allocation to the belowground microbial community has been shown to enhance the decomposition of recalcitrant C compounds (Ekberg *et al.*, 2007) and to result in a long-term net C loss from soil (Carney *et al.*, 2007; Dijkstra & Cheng, 2007). Carney *et al.* (2007) showed that elevated root C input altered the microbial community structure towards a higher proportion of fungi, which in turn led to greater priming of SOM decomposition by leaf litter. This indicates that SOM decomposition may be determined by microbial community composition *and* an appropriate source of energy. In the present study, we compared a system with a specialized root-C-based microbial community to a system inhabited by a community not supported by tree root C. In the ‘intact’ system, we see clear seasonal trends, suggesting that FOM degradation, as indicated by actual cellulase/amylase and protease activities, seems to be enhanced in autumn, whereas SOM degradation, as indicated by a higher potential activity of oxidative enzymes, may be enhanced during the summer months. In the girdled system, however, this trend disappeared and lower rates for cellulase and protein degradation, especially in autumn, were found. The possible conclusion that mycorrhizal fungi may be involved in autumn litter degradation, however, still needs to be confirmed by direct measurements.

Overall, our study demonstrated the importance of plant–decomposer interactions for C and N cycling in temperate forests on a seasonal time-scale. Our results showed that extracellular enzymes mediating the decomposition of SOM and litter in a beech forest soil are linked to microbial community composition and exhibit a strong seasonal pattern. Our findings indicate that, through belowground C allocation, trees alter microbial community structure and thus may affect the seasonal pattern of microbial decomposition processes.

## Supporting Information

Fig. S1 Leaf litter fall collected by litter traps in the second sampling year (August–December 2007) in control and girdled plots.

Fig. S2 Microbial community composition of bimonthly samplings on the first two axes of the canonical correspondence analysis.

Table S1 Phospholipid fatty acids of different microbial groups in control, girdled and fertilized soils over a time period of 2 yr.

Table S2 Linear regressions of phospholipid fatty acid biomarkers and soil parameters for control plots only, girdled plots only, and both control and girdled plots together.
