# Supplementary material for: Belowground carbon allocation by trees drives seasonal patterns of extracellular enzyme activities by altering microbial community composition in a beech forest soil
Source: New Phytol. 2010 Aug;187(3):843–58. doi: 10.1111/j.1469-8137.2010.03321.x (PMC2916209; doi:10.1111/j.1469-8137.2010.03321.x)
Supplement: Supplementary file 1 [file nph0187-0843-SD1.doc]

**Supporting Information Fig. S1** Leaf litterfall collected by litter traps in the second sampling year (August–December 2007) in control and girdled plots. Black bars, controls; grey bars, girdled plots. Bars represent means of six litter traps (1 m × 0.5 m) each installed in one of six control and girdled plots, respectively. Error bars indicate 1 SE (*n* = 6).

**Fig. S2** Microbial community composition of bi-monthly samplings on the first two axes of the canonical correspondence analysis (CCA; see Fig. 6). The first axis displays the variability of community data due to seasonal variation (a), whereas the second axis separates the girdling treatment from control and fertilised treatment (b). Grey circles, girdled plots; black squares, control plots; open triangles, fertilized plots. All data are plotted on the actual sampling date; ticks are for the 15^th^ day of each month. Error bars indicate 1 SE (*n* = 6).
