# Supplementary material for: Belowground carbon allocation by trees drives seasonal patterns of extracellular enzyme activities by altering microbial community composition in a beech forest soil
Source: New Phytol. 2010 Aug;187(3):843–58. doi: 10.1111/j.1469-8137.2010.03321.x (PMC2916209; doi:10.1111/j.1469-8137.2010.03321.x)
Supplement: Supplementary file 2 [file nph0187-0843-SD2.doc]

**Supporting Information Table S1** Phospholipid fatty acids (nmol g^−1^ dry soil) of different microbial groups in control, girdled and fertilised soils over a time period of 2 yr

| year | mon | day | total PLFA | | | | | | bacteria | | | | | | | |
| --- | --- | --- | --- | --- | --- | --- | --- | --- | --- | --- | --- | --- | --- | --- | --- | --- |
|  |  |  | C | | G | | F | | C | | G | | F | | | |
|  |  |  | mean | se | mean | se | mean | se | mean | se | mean | se | mean | | se | |
| *2006* | *Jun* | *6* | 337.7 | 34.4 | 395.7 | 20.4 | 410.5 | 33.7 | 164.4 | 16.2 | 189.0 | 8.6 | 181.8 | | 16.8 | |
| *2006* | *Jul* | *31* | 501.9 | 17.6 | 432.4 | 22.1 | 508.3 | 47.0 | 228.4 | 9.4 | 202.2 | 10.0 | 235.6 | | 19.5 | |
| *2006* | *Oct* | *3* | 497.4 | 21.2 | 481.7 | 22.5 | 499.9 | 45.1 | 228.9 | 10.1 | 230.4 | 10.8 | 233.9 | | 20.7 | |
| *2006* | *Dec* | *4* | 438.1 | 22.7 | 369.5 | 24.6 | 453.8 | 26.0 | 186.4 | 10.2 | 167.3 | 11.9 | 204.6 | | 11.7 | |
| *2007* | *Feb* | *12* | 242.4 | 21.4 | 239.4 | 18.3 | 230.7 | 24.0 | 97.6 | 10.7 | 94.2 | 8.8 | 88.8 | | 10.5 | |
| *2007* | *Apr* | *17* | 423.9 | 25.0 | 350.5 | 30.4 | 430.0 | 42.7 | 198.5 | 11.6 | 166.6 | 14.1 | 201.0 | | 19.7 | |
| *2007* | *Jun* | *18* | 505.9 | 31.5 | 424.1 | 29.5 | 469.9 | 44.9 | 217.5 | 13.5 | 186.2 | 13.9 | 195.9 | | 22.4 | |
| *2007* | *Jul* | *24* | 276.2 | 30.5 | 229.9 | 40.2 | 300.1 | 20.9 | 105.9 | 15.1 | 86.7 | 19.4 | 109.8 | | 11.6 | |
| *2007* | *Sep* | *24* | 448.4 | 75.2 | 317.7 | 18.8 | 405.3 | 33.1 | 178.8 | 31.9 | 132.3 | 8.4 | 168.2 | | 15.4 | |
| *2007* | *Nov* | *20* | 351.9 | 36.8 | 299.6 | 12.4 | 390.3 | 50.2 | 130.8 | 23.1 | 107.8 | 6.8 | 149.8 | | 18.9 | |
| *2008* | *Jan* | *15* | 356.4 | 52.4 | 264.8 | 29.1 | 306.5 | 28.2 | 146.7 | 25.2 | 106.6 | 12.0 | 121.6 | | 14.7 | |
| *2008* | *Mar* | *10* | 250.0 | 22.5 | 256.1 | 23.7 | 317.1 | 22.5 | 96.9 | 11.0 | 98.6 | 11.2 | 128.5 | | 8.5 | |
| *2008* | *May* | *13* | 383.8 | 41.2 | 404.2 | 32.1 | 434.4 | 48.3 | 148.8 | 17.8 | 161.7 | 14.8 | 166.5 | | 20.1 | |
|  | *mean* |  | **385.7^a^** | 25.8 | **343.5^b^** | 22.8 | **396.7 ^a^** | 23.5******* | **163.8 ^a^** | 13.2 | **148.4 ^b^** | 13.0 | **168.2 ^a^** | | 12.9** | |
|  |  |  |  | | | | | |  | | | | | | | |
|  |  |  | gram negative bacteria | | | | | | gram positive bacteria | | | | | | | |
|  |  |  | C | | G | | F | | C | | G | | F | | | |
|  |  |  | mean | se | mean | se | mean | se | mean | se | mean | se | mean | | se | |
| *2006* | *Jun* | *6* | 85.4 | 7.2 | 100.4 | 5.3 | 114.2 | 10.1 | 79.8 | 9.6 | 88.0 | 3.8 | 68.3 | | 7.7 | |
| *2006* | *Aug* | *31* | 108.4 | 4.0 | 98.0 | 5.3 | 122.1 | 13.1 | 117.0 | 6.1 | 101.7 | 4.8 | 112.1 | | 7.7 | |
| *2006* | *Oct* | *3* | 112.4 | 6.1 | 111.8 | 5.9 | 119.5 | 14.2 | 115.1 | 4.6 | 116.2 | 4.8 | 113.9 | | 7.7 | |
| *2006* | *Dec* | *4* | 106.7 | 7.2 | 87.1 | 5.7 | 101.6 | 5.8 | **77.4^a^** | 3.6 | **77.8^a^** | 8.1 | **103.0^b^** | | 6.6* | |
| *2007* | *Feb* | *12* | 66.3 | 6.3 | 61.6 | 5.8 | 61.7 | 6.3 | 29.7 | 4.4 | 30.6 | 4.4 | 24.9 | | 4.9 | |
| *2007* | *Apr* | *17* | 102.6 | 7.2 | 82.8 | 8.3 | 101.2 | 11.7 | 96.6 | 4.6 | 82.0 | 6.7 | 99.1 | | 8.4 | |
| *2007* | *Jun* | *18* | 117.4 | 7.5 | 96.8 | 7.1 | 117.9 | 15.7 | 98.0 | 6.0 | 86.6 | 7.2 | 75.3 | | 7.3 | |
| *2007* | *Jul* | *24* | 64.9 | 8.2 | 52.6 | 8.2 | 66.1 | 4.7 | 37.3 | 7.8 | 31.4 | 11.5 | 39.6 | | 8.1 | |
| *2007* | *Sep* | *24* | 99.1 | 14.7 | 73.8 | 4.8 | 89.9 | 11.5 | 76.5 | 17.1 | 56.8 | 5.5 | 75.9 | | 8.9 | |
| *2007* | *Nov* | *20* | 91.9 | 12.8 | 79.5 | 3.5 | 99.4 | 10.7 | 34.4 | 11.1 | 24.3 | 4.5 | 44.9 | | 8.6 | |
| *2008* | *Jan* | *15* | 88.7 | 12.1 | 68.4 | 7.8 | 77.4 | 8.5 | 56.4 | 13.9 | 36.8 | 7.3 | 42.7 | | 7.4 | |
| *2008* | *Mar* | *10* | 67.7 | 7.8 | 64.8 | 6.0 | 83.9 | 5.3 | 27.7 | 5.4 | 31.6 | 6.8 | 45.0 | | 5.1 | |
| *2008* | *May* | *13* | 99.2 | 10.8 | 103.7 | 8.9 | 116.0 | 11.8 | 47.7 | 8.6 | 56.9 | 9.0 | 47.0 | | 10.7 | |
|  | *mean* |  | **93.1 ^a^** | 4.9 | **83.2 ^b^** | 5.1 | **97.8 ^a^** | 5.7*** | 68.7 | 8.9 | 63.1 | 8.5 | 68.6 | | 8.5 | |
|  |  |  |  | | | | | |  | | | | | | | |
|  |  |  | fungi ( 18:2ω6,9) | | | | | | actinomycetes | | | | | | | |
|  |  |  | C | | G | | F | | C | | G | | F | | | |
|  |  |  | mean | se | mean | se | mean | se | mean | se | mean | se | mean | | se | |
| *2006* | *Jun* | *6* | 15.4 | 1.4 | 15.6 | 1.6 | 23.0 | 3.9 | 15.4 | 2.1 | 16.0 | 0.8 | 17.0 | | 1.2 | |
| *2006* | *Aug* | *31* | 24.0 | 1.6 | 16.2 | 1.3 | 26.0 | 5.1 | 23.0 | 1.7 | 21.4 | 1.1 | 24.6 | | 1.9 | |
| *2006* | *Oct* | *3* | **26.5^a^** | 2.0 | **14.3^b^** | 1.0 | **24.6^a^** | 3.3** | 23.6 | 1.2 | 23.0 | 1.1 | 25.5 | | 2.3 | |
| *2006* | *Dec* | *4* | **29.1^a^** | 4.1 | **10.7^b^** | 1.0 | **21.2^a^** | 2.0*** | 24.6 | 1.4 | 23.5 | 1.3 | 31.2 | | 4.8 | |
| *2007* | *Feb* | *12* | 13.7 | 1.0 | 11.5 | 1.9 | 14.9 | 2.0 | 13.8 | 1.6 | 19.3 | 3.8 | 16.0 | | 1.8 | |
| *2007* | *Apr* | *17* | **21.1^a^** | 1.5 | **10.2^b^** | 1.1 | **20.7^a^** | 2.4*** | 25.2 | 0.7 | 22.0 | 2.2 | 26.7 | | 2.5 | |
| *2007* | *Jun* | *18* | **29.1^a^** | 3.1 | **11.0^b^** | 1.0 | **29.3^a^** | 3.9* | 30.9 | 2.1 | 28.7 | 3.6 | 29.5 | | 2.2 | |
| *2007* | *Jul* | *24* | **14.7^a^** | 1.5 | **6.2^b^** | 0.9 | **14.5^a^** | 1.0* | 18.4 | 3.4 | 15.6 | 4.0 | 19.6 | | 2.8 | |
| *2007* | *Sep* | *24* | **29.4^a^** | 7.4 | **8.3^b^** | 1.0 | **19.7^ab^** | 2.5* | 36.2 | 5.8 | 27.4 | 1.5 | 35.4 | | 4.2 | |
| *2007* | *Nov* | *20* | **17.1^a^** | 2.6 | **7.0^b^** | 1.5 | **20.7^a^** | 3.2** | 19.2 | 2.9 | 18.2 | 2.3 | 26.3 | | 3.6 | |
| *2008* | *Jan* | *15* | **17.7^a^** | 1.7 | **7.2^b^** | 1.2 | **14.0^a^** | 1.1* | 27.4 | 4.6 | 21.2 | 2.5 | 22.6 | | 1.0 | |
| *2008* | *Mar* | *10* | 10.1 | 2.1 | 10.4 | 2.3 | 14.5 | 1.7 | 17.8 | 1.6 | 19.8 | 2.1 | 23.5 | | 3.1 | |
| *2008* | *May* | *13* | 24.7 | 3.2 | 13.5 | 1.7 | 28.8 | 3.9 | 29.8 | 4.4 | 35.1 | 3.7 | 31.8 | | 3.6 | |
|  | *mean* |  | **21.0 ^a^** | 1.8 | **10.9 ^b^** | 0.9 | **20.9 ^a^** | 1.5*** | **23.5^ab^** | 1.8 | **22.4^a^** | 1.5 | **25.3^b^** | | 1.6* | |
|  |  |  |  | | | | | |  | | | | | | | |
|  |  |  | plant & fungi (18:3ω3,6,9) | | | | | | fungi (18:1ω9c) | | | | | | | |
|  |  |  | C | | G | | F | | C | | G | | | F | | |
|  |  |  | mean | se | mean | se | mean | se | mean | se | mean | se | | mean | | se |
| *2006* | *Jun* | *6* | 1.3 | 0.3 | 2.1 | 0.4 | 2.2 | 0.7 | **25.9^b^** | 3.6 | **35.6^ab^** | 2.6 | | **40.7^a^** | | 2.8* |
| *2006* | *Aug* | *31* | 2.3 | 0.3 | 1.2 | 0.5 | 2.7 | 0.5 | 41.5 | 2.4 | 37.7 | 2.0 | | 38.4 | | 8.5 |
| *2006* | *Oct* | *3* | **1.8^ab^** | 0.3 | **0.5^b^** | 0.2 | **2.4^a^** | 0.5* | 42.9 | 3.1 | 41.2 | 1.9 | | 42.7 | | 3.9 |
| *2006* | *Dec* | *4* | **2.3^a^** | 0.3 | **0.5^b^** | 0.1 | **2.6^a^** | 0.2*** | **43.7^a^** | 3.2 | **32.6^b^** | 2.4 | | **39.9^ab^** | | 2.8* |
| *2007* | *Feb* | *12* | 1.1 | 0.3 | 0.6 | 0.2 | 1.1 | 0.4 | 28.3 | 2.7 | 25.3 | 1.8 | | 27.8 | | 2.7 |
| *2007* | *Apr* | *17* | 1.8 | 0.2 | 1.6 | 0.2 | 2.1 | 0.3 | 35.7 | 2.5 | 29.0 | 2.8 | | 37.6 | | 4.7 |
| *2007* | *Jun* | *18* | **2.6^a^** | 0.2 | **1.6^b^** | 0.1 | **3.0^a^** | 0.4** | 46.2 | 3.1 | 39.8 | 3.5 | | 48.1 | | 4.2 |
| *2007* | *Jul* | *24* | **1.0^ab^** | 0.2 | **0.4^b^** | 0.2 | **1.0^a^** | 0.1* | 25.2 | 4.9 | 24.1 | 3.5 | | 35.3 | | 2.2 |
| *2007* | *Sep* | *24* | 1.9 | 0.5 | 1.1 | 0.2 | 0.7 | 0.2 | 43.8 | 5.6 | 29.8 | 2.0 | | 39.3 | | 2.7 |
| *2007* | *Nov* | *20* | 1.6 | 0.2 | 1.2 | 0.2 | 2.1 | 0.4 | 47.1 | 4.3 | 39.1 | 2.1 | | 48.1 | | 4.9 |
| *2008* | *Jan* | *15* | **1.3^a^** | 0.2 | **0.2^b^** | 0.2 | **0.9^ab^** | 0.3** | 39.3 | 4.9 | 28.7 | 3.5 | | 33.4 | | 3.0 |
| *2008* | *Mar* | *10* | 0.7 | 0.3 | 0.6 | 0.4 | 0.6 | 0.3 | 29.5 | 2.1 | 28.4 | 2.4 | | 34.7 | | 2.9 |
| *2008* | *May* | *13* | **1.5^ab^** | 0.3 | **1.0^a^** | 0.3 | **3.2^b^** | 0.7* | 42.2 | 4.9 | 41.1 | 4.0 | | 52.5 | | 6.2 |
|  | *mean* |  | **1.8 ^a^** | 0.2 | **1.0^b^** | 0.2 | **1.9 ^a^** | 0.3*** | **37.8 ^a^** | 2.2 | **33.3 ^b^** | 1.7 | | **39.9 ^a^** | | 1.9*** |

Bold letters indicate statistical significant differences among treatments (C, Control; G, Girdling; F, Fertilisation) for each month and microbial group; levels of significance are given in the last column (***, *P* < 0.001; **, *P* < 0.01; *, *P* < 0.05, tested by ANOVA). Different superscript letters indicate statistical significant differences between specific treatments (Tukey HSD; *P* < 0.05). The statistical difference between treatments over all samplings (assessed by ANCOVA with sampling month as covariate) is indicated for each microbial group together with the overall mean.
