# Supplementary material for: Belowground carbon allocation by trees drives seasonal patterns of extracellular enzyme activities by altering microbial community composition in a beech forest soil
Source: New Phytol. 2010 Aug;187(3):843–58. doi: 10.1111/j.1469-8137.2010.03321.x (PMC2916209; doi:10.1111/j.1469-8137.2010.03321.x)
Supplement: Supplementary file 3 [file nph0187-0843-SD3.doc]

**Supporting Information Table S2** Linear regressions of phospholipid fatty acid biomarkers and soil parameters for control plots only, girdled plots only and both, control and girdled plots together

|  |  | Control plots only | | | | | | | | | | | |  | Girdling plots only | | | | | | | | | | | |  | Control and Girdling | | | | | | | | | | | |
| --- | --- | --- | --- | --- | --- | --- | --- | --- | --- | --- | --- | --- | --- | --- | --- | --- | --- | --- | --- | --- | --- | --- | --- | --- | --- | --- | --- | --- | --- | --- | --- | --- | --- | --- | --- | --- | --- | --- | --- |
|  |  | Wat. content | Soil temp | Cellobiosid. | Chitinase | N-Acetyl-g. | Leu-pept | Phenolox. | Perox. | Act. prot | Gluc prod. | dN | DOC: dN |  | Wat. content | Soil temp | Cellobios. | Chitinase | N-Acetyl. | Leu-pept | Phenolox. | Perox. | Act. prot | Gluc prod. | dN | DOC: dN |  | Wat. content | Soil temp | Cellobiosid. | Chitinase | N-Acetyl-g. | Leu-pept | Phenolox. | Perox. | Act. prot | Gluc prod. | dN | DOC: dN |
| gram + | *i15:0* | 0.19 | 0.41 | 0.28 | 0.18 | 0.19 | 0.49 | 0.37 | **0.60** | 0.03 | 0.15 | 0.08 | 0.00 |  | 0.14 | 0.31 | 0.13 | 0.22 | 0.17 | **0.56** | 0.37 | **0.56** | 0.27 | 0.00 | 0.29 | 0.21 |  | 0.16 | 0.36 | 0.21 | 0.17 | 0.18 | **0.52** | 0.37 | **0.56** | 0.10 | 0.05 | 0.08 | 0.01 |
|  | *a15:0* | 0.12 | 0.30 | 0.26 | 0.18 | 0.19 | 0.47 | 0.28 | 0.45 | 0.05 | 0.16 | 0.06 | 0.00 |  | 0.15 | 0.31 | 0.19 | 0.24 | 0.20 | **0.55** | 0.42 | **0.56** | 0.27 | 0.00 | 0.20 | 0.16 |  | 0.14 | 0.30 | 0.23 | 0.20 | 0.19 | 0.50 | 0.33 | 0.47 | 0.14 | 0.08 | 0.03 | 0.00 |
|  | *i16:0* | 0.23 | 0.43 | 0.28 | 0.21 | 0.13 | 0.35 | 0.32 | 0.45 | 0.03 | 0.20 | 0.06 | 0.00 |  | 0.09 | 0.34 | 0.17 | 0.27 | 0.17 | 0.38 | 0.47 | 0.49 | 0.28 | 0.00 | 0.29 | 0.18 |  | 0.16 | 0.38 | 0.23 | 0.21 | 0.15 | 0.36 | 0.38 | 0.45 | 0.11 | 0.09 | 0.06 | 0.00 |
|  | *i17:0* | 0.24 | 0.42 | 0.26 | 0.21 | 0.05 | 0.20 | 0.21 | 0.26 | 0.01 | 0.17 | 0.03 | 0.01 |  | 0.05 | 0.31 | 0.12 | 0.16 | 0.08 | 0.30 | 0.37 | 0.34 | 0.21 | 0.01 | 0.34 | 0.19 |  | 0.13 | 0.36 | 0.19 | 0.18 | 0.06 | 0.25 | 0.27 | 0.29 | 0.07 | 0.08 | 0.07 | 0.00 |
|  | *a17:0* | 0.18 | 0.33 | 0.22 | 0.18 | 0.03 | 0.14 | 0.09 | 0.10 | 0.03 | 0.18 | 0.00 | 0.05 |  | 0.05 | 0.25 | 0.25 | 0.19 | 0.07 | 0.23 | 0.44 | 0.39 | 0.12 | 0.03 | 0.18 | 0.07 |  | 0.14 | 0.27 | 0.23 | 0.22 | 0.05 | 0.16 | 0.18 | 0.18 | 0.12 | 0.17 | 0.00 | 0.05 |
|  | *10me16* | 0.03 | 0.08 | 0.13 | 0.17 | 0.00 | 0.01 | 0.03 | 0.01 | 0.00 | 0.00 | 0.02 | 0.01 |  | 0.04 | 0.02 | 0.01 | 0.04 | 0.01 | 0.04 | 0.04 | 0.01 | 0.21 | 0.05 | 0.15 | 0.07 |  | 0.00 | 0.05 | 0.06 | 0.11 | 0.00 | 0.02 | 0.03 | 0.00 | 0.04 | 0.00 | 0.01 | 0.00 |
| gram - | *18:1w7* | 0.01 | 0.02 | 0.08 | 0.08 | 0.00 | 0.03 | 0.01 | 0.00 | 0.03 | 0.07 | 0.04 | 0.06 |  | 0.03 | 0.02 | 0.11 | 0.10 | 0.07 | 0.13 | 0.30 | 0.06 | 0.09 | 0.00 | 0.05 | 0.00 |  | 0.00 | 0.02 | 0.09 | 0.11 | 0.01 | 0.06 | 0.08 | 0.01 | 0.09 | 0.06 | 0.00 | 0.06 |
|  | *cy17:0* | 0.03 | 0.03 | 0.04 | 0.07 | 0.01 | 0.03 | 0.01 | 0.00 | 0.00 | 0.02 | 0.02 | 0.02 |  | 0.02 | 0.02 | 0.03 | 0.09 | 0.01 | 0.15 | 0.20 | 0.00 | 0.10 | 0.13 | 0.08 | 0.02 |  | 0.00 | 0.02 | 0.03 | 0.09 | 0.00 | 0.07 | 0.06 | 0.00 | 0.05 | 0.00 | 0.00 | 0.02 |
|  | *16:1w7* | 0.08 | 0.20 | 0.23 | 0.14 | 0.31 | 0.39 | 0.27 | **0.53** | 0.20 | 0.41 | 0.04 | 0.02 |  | 0.12 | 0.17 | 0.22 | 0.23 | 0.23 | 0.42 | 0.35 | **0.60** | 0.24 | 0.02 | 0.13 | 0.07 |  | 0.11 | 0.18 | 0.22 | 0.16 | 0.27 | 0.40 | 0.30 | **0.54** | 0.22 | 0.22 | 0.02 | 0.00 |
|  | *16:1w9* | 0.24 | 0.41 | 0.16 | 0.13 | 0.11 | 0.45 | 0.17 | 0.28 | 0.09 | 0.15 | 0.03 | 0.02 |  | 0.18 | 0.33 | 0.16 | 0.19 | 0.18 | **0.56** | 0.43 | 0.48 | 0.33 | 0.01 | 0.22 | 0.20 |  | 0.22 | 0.36 | 0.15 | 0.15 | 0.14 | 0.49 | 0.27 | 0.36 | 0.21 | 0.07 | 0.03 | 0.00 |
|  | *cy18:0* | 0.07 | 0.19 | 0.16 | 0.12 | 0.06 | 0.26 | 0.31 | 0.30 | 0.04 | 0.09 | 0.03 | 0.00 |  | 0.01 | 0.07 | 0.09 | 0.18 | 0.09 | 0.43 | 0.44 | 0.26 | 0.24 | 0.08 | 0.19 | 0.07 |  | 0.04 | 0.12 | 0.13 | 0.15 | 0.07 | 0.32 | 0.34 | 0.25 | 0.13 | 0.02 | 0.02 | 0.01 |
|  | *cy19:0* | 0.35 | 0.28 | 0.19 | 0.17 | 0.06 | 0.13 | 0.00 | 0.15 | 0.06 | **0.62** | 0.00 | 0.22 |  | 0.03 | 0.01 | 0.01 | 0.01 | 0.01 | 0.22 | 0.06 | 0.27 | 0.11 | 0.10 | 0.42 | 0.14 |  | 0.11 | 0.10 | 0.04 | 0.04 | 0.03 | 0.16 | 0.02 | 0.21 | 0.03 | 0.24 | 0.18 | 0.00 |
|  | *16:1w5* | 0.08 | 0.26 | 0.35 | 0.32 | 0.22 | 0.33 | 0.28 | 0.37 | 0.11 | 0.20 | 0.01 | 0.04 |  | 0.05 | 0.19 | 0.23 | 0.30 | 0.22 | 0.46 | **0.51** | 0.39 | 0.35 | 0.02 | 0.17 | 0.10 |  | 0.10 | 0.20 | 0.28 | 0.36 | 0.20 | 0.35 | 0.31 | 0.29 | 0.28 | 0.14 | 0.00 | 0.06 |
| bact | *18:1w5* | 0.07 | 0.09 | 0.14 | 0.13 | 0.00 | 0.02 | 0.00 | 0.00 | 0.03 | 0.21 | 0.03 | 0.14 |  | 0.07 | 0.13 | 0.13 | 0.13 | 0.09 | 0.33 | **0.52** | **0.50** | 0.16 | 0.02 | 0.16 | 0.08 |  | 0.12 | 0.07 | 0.11 | 0.23 | 0.02 | 0.06 | 0.04 | 0.04 | 0.22 | 0.27 | 0.08 | 0.27 |
|  | *17:00* | 0.17 | 0.27 | 0.15 | 0.17 | 0.02 | 0.08 | 0.11 | 0.12 | 0.01 | 0.15 | 0.02 | 0.02 |  | 0.00 | 0.13 | 0.08 | 0.06 | 0.02 | 0.16 | 0.31 | 0.10 | 0.12 | 0.01 | 0.16 | 0.08 |  | 0.08 | 0.20 | 0.12 | 0.15 | 0.02 | 0.10 | 0.17 | 0.09 | 0.06 | 0.08 | 0.01 | 0.02 |
|  | *15:00* | 0.26 | 0.45 | 0.23 | 0.19 | 0.16 | 0.37 | 0.31 | **0.52** | 0.03 | 0.23 | 0.11 | 0.00 |  | 0.14 | 0.28 | 0.15 | 0.27 | 0.23 | 0.46 | 0.42 | **0.64** | 0.28 | 0.01 | 0.25 | 0.16 |  | 0.20 | 0.36 | 0.19 | 0.21 | 0.19 | 0.41 | 0.35 | **0.55** | 0.12 | 0.12 | 0.05 | 0.00 |
|  | *17:1w6* | 0.15 | 0.21 | 0.15 | 0.09 | 0.00 | 0.16 | 0.09 | 0.11 | 0.00 | 0.13 | 0.01 | 0.01 |  | 0.01 | 0.14 | 0.14 | 0.12 | 0.06 | 0.35 | 0.41 | 0.28 | 0.09 | 0.00 | 0.19 | 0.06 |  | 0.07 | 0.17 | 0.15 | 0.10 | 0.02 | 0.24 | 0.21 | 0.18 | 0.04 | 0.07 | 0.03 | 0.00 |
| fungi | *18:2w6* | 0.15 | 0.20 | 0.12 | 0.21 | 0.08 | 0.21 | 0.02 | 0.06 | 0.18 | 0.26 | 0.01 | 0.15 |  | 0.08 | 0.07 | 0.09 | 0.11 | 0.07 | 0.26 | **0.54** | 0.25 | 0.29 | 0.07 | 0.05 | 0.07 |  | 0.17 | 0.08 | 0.08 | 0.29 | 0.05 | 0.12 | 0.04 | 0.03 | 0.36 | 0.27 | 0.15 | 0.35 |
|  | *18:1w9* | 0.01 | 0.02 | 0.08 | 0.08 | 0.00 | 0.04 | 0.00 | 0.00 | 0.04 | 0.12 | 0.04 | 0.11 |  | 0.00 | 0.06 | 0.13 | 0.11 | 0.06 | 0.17 | 0.42 | 0.19 | 0.07 | 0.01 | 0.08 | 0.01 |  | 0.01 | 0.03 | 0.10 | 0.12 | 0.01 | 0.08 | 0.07 | 0.04 | 0.11 | 0.12 | 0.00 | 0.09 |
| f&p | *18:3w3* | 0.09 | 0.05 | 0.05 | 0.17 | 0.07 | 0.07 | 0.01 | 0.01 | 0.23 | **0.57** | 0.00 | 0.24 |  | 0.01 | 0.05 | **0.64** | 0.15 | 0.17 | 0.01 | 0.20 | 0.11 | 0.00 | 0.20 | 0.07 | 0.09 |  | 0.06 | 0.04 | 0.14 | 0.26 | 0.08 | 0.01 | 0.00 | 0.01 | 0.27 | **0.57** | 0.17 | 0.38 |
| general | *14:00* | 0.23 | 0.43 | 0.15 | 0.07 | 0.10 | 0.48 | 0.36 | **0.57** | 0.00 | 0.08 | 0.17 | 0.02 |  | 0.19 | 0.29 | 0.12 | 0.17 | 0.16 | **0.63** | 0.41 | **0.63** | 0.22 | 0.00 | 0.19 | 0.17 |  | 0.21 | 0.36 | 0.14 | 0.09 | 0.13 | **0.55** | 0.38 | **0.58** | 0.06 | 0.03 | 0.05 | 0.01 |
|  | *16:00* | 0.24 | 0.39 | 0.24 | 0.22 | 0.11 | 0.27 | 0.22 | 0.34 | 0.05 | 0.27 | 0.04 | 0.03 |  | 0.08 | 0.28 | 0.15 | 0.21 | 0.13 | 0.41 | 0.48 | 0.46 | 0.28 | 0.00 | 0.30 | 0.18 |  | 0.17 | 0.33 | 0.20 | 0.23 | 0.12 | 0.32 | 0.30 | 0.36 | 0.15 | 0.15 | 0.03 | 0.01 |
|  | *16:1w11* | 0.08 | 0.24 | 0.35 | 0.28 | 0.17 | 0.37 | 0.25 | 0.35 | 0.06 | 0.16 | 0.01 | 0.02 |  | 0.04 | 0.18 | 0.21 | 0.37 | 0.25 | **0.50** | 0.46 | 0.41 | 0.36 | 0.01 | 0.23 | 0.10 |  | 0.08 | 0.20 | 0.28 | 0.32 | 0.20 | 0.41 | 0.31 | 0.33 | 0.21 | 0.09 | 0.01 | 0.02 |
|  | *16:1w6* | 0.27 | 0.42 | 0.27 | 0.28 | 0.13 | 0.30 | 0.12 | 0.25 | 0.07 | 0.28 | 0.01 | 0.06 |  | 0.10 | 0.35 | 0.20 | 0.25 | 0.16 | 0.43 | 0.43 | **0.52** | 0.27 | 0.01 | 0.31 | 0.19 |  | 0.19 | 0.37 | 0.23 | 0.27 | 0.14 | 0.35 | 0.23 | 0.35 | 0.19 | 0.17 | 0.03 | 0.02 |
|  | *17:1w6* | 0.16 | 0.28 | 0.20 | 0.22 | 0.06 | 0.22 | 0.20 | 0.20 | 0.03 | 0.11 | 0.00 | 0.02 |  | 0.00 | 0.17 | 0.11 | 0.17 | 0.13 | 0.29 | 0.29 | 0.24 | 0.33 | 0.00 | 0.34 | 0.17 |  | 0.05 | 0.22 | 0.15 | 0.17 | 0.09 | 0.25 | 0.24 | 0.21 | 0.11 | 0.04 | 0.08 | 0.00 |
|  | *18:00* | 0.26 | 0.36 | 0.11 | 0.14 | 0.08 | 0.16 | 0.16 | 0.28 | 0.05 | 0.41 | 0.10 | 0.03 |  | 0.05 | 0.18 | 0.07 | 0.04 | 0.02 | 0.26 | 0.33 | 0.47 | 0.09 | 0.05 | 0.25 | 0.14 |  | 0.17 | 0.26 | 0.10 | 0.14 | 0.06 | 0.18 | 0.18 | 0.28 | 0.09 | 0.28 | 0.01 | 0.02 |
| groups | *gram+* | 0.19 | 0.40 | 0.29 | 0.20 | 0.17 | 0.44 | 0.32 | 0.49 | 0.04 | 0.18 | 0.06 | 0.00 |  | 0.13 | 0.33 | 0.17 | 0.24 | 0.17 | **0.51** | 0.43 | **0.55** | 0.27 | 0.00 | 0.27 | 0.19 |  | 0.16 | 0.36 | 0.23 | 0.20 | 0.17 | 0.47 | 0.36 | 0.50 | 0.12 | 0.08 | 0.06 | 0.00 |
|  | *gram-* | 0.06 | 0.11 | 0.18 | 0.16 | 0.04 | 0.16 | 0.08 | 0.09 | 0.08 | 0.18 | 0.01 | 0.07 |  | 0.00 | 0.08 | 0.18 | 0.20 | 0.14 | 0.33 | 0.48 | 0.24 | 0.20 | 0.00 | 0.13 | 0.03 |  | 0.03 | 0.09 | 0.18 | 0.19 | 0.08 | 0.23 | 0.22 | 0.14 | 0.17 | 0.10 | 0.00 | 0.04 |
|  | *bact* | 0.15 | 0.32 | 0.28 | 0.22 | 0.12 | 0.36 | 0.24 | 0.34 | 0.06 | 0.21 | 0.02 | 0.02 |  | 0.06 | 0.24 | 0.20 | 0.25 | 0.18 | 0.49 | **0.52** | 0.48 | 0.27 | 0.00 | 0.24 | 0.12 |  | 0.11 | 0.27 | 0.24 | 0.23 | 0.14 | 0.41 | 0.34 | 0.38 | 0.16 | 0.11 | 0.03 | 0.01 |
|  | *fungi* | 0.06 | 0.07 | 0.09 | 0.14 | 0.02 | 0.11 | 0.00 | 0.01 | 0.12 | 0.21 | 0.03 | 0.15 |  | 0.01 | 0.07 | 0.13 | 0.13 | 0.08 | 0.23 | **0.53** | 0.24 | 0.13 | 0.00 | 0.08 | 0.02 |  | 0.07 | 0.06 | 0.10 | 0.22 | 0.03 | 0.12 | 0.07 | 0.04 | 0.25 | 0.22 | 0.05 | 0.22 |
|  | *all* | 0.11 | 0.32 | 0.23 | 0.32 | 0.26 | 0.31 | 0.11 | 0.30 | 0.12 | 0.17 | 0.04 | 0.04 |  | 0.02 | 0.25 | 0.15 | 0.31 | 0.39 | **0.52** | 0.45 | **0.53** | 0.34 | 0.00 | 0.20 | 0.11 |  | 0.09 | 0.26 | 0.20 | 0.35 | 0.30 | 0.37 | 0.20 | 0.34 | 0.26 | 0.13 | 0.00 | 0.04 |
| processes | *cellob.* | 0.01 | 0.10 | **1.00** | **0.54** | 0.40 | 0.05 | 0.32 | 0.42 | 0.02 | 0.21 | 0.00 | 0.02 |  | 0.05 | 0.03 | **1.00** | 0.30 | 0.21 | 0.01 | 0.10 | 0.06 | 0.00 | 0.07 | 0.08 | 0.14 |  | 0.02 | 0.06 | **1.00** | 0.42 | 0.32 | 0.03 | 0.20 | 0.20 | 0.02 | 0.18 | 0.03 | 0.03 |
|  | *chitin.* | 0.00 | 0.07 | **0.54** | **1.00** | **0.71** | 0.01 | 0.13 | 0.20 | 0.15 | 0.37 | 0.00 | 0.09 |  | 0.05 | 0.00 | 0.30 | **1.00** | **0.71** | 0.10 | 0.30 | 0.11 | 0.21 | 0.03 | 0.01 | 0.05 |  | 0.00 | 0.02 | 0.42 | **1.00** | **0.62** | 0.03 | 0.13 | 0.10 | 0.25 | 0.35 | 0.05 | 0.19 |
|  | *N-acet.* | 0.01 | 0.05 | 0.40 | **0.71** | **1.00** | 0.11 | 0.14 | 0.32 | 0.42 | 0.34 | 0.00 | 0.12 |  | 0.06 | 0.00 | 0.21 | **0.71** | **1.00** | 0.21 | 0.15 | 0.17 | 0.48 | 0.06 | 0.03 | 0.00 |  | 0.02 | 0.02 | 0.32 | **0.62** | **1.00** | 0.15 | 0.14 | 0.23 | 0.37 | 0.21 | 0.00 | 0.04 |
|  | *L-pept* | 0.10 | 0.13 | 0.05 | 0.01 | 0.11 | **1.00** | 0.07 | 0.30 | 0.10 | 0.00 | 0.02 | 0.00 |  | 0.03 | 0.06 | 0.01 | 0.10 | 0.21 | **1.00** | 0.12 | 0.31 | 0.46 | 0.07 | 0.32 | 0.25 |  | 0.05 | 0.09 | 0.03 | 0.03 | 0.15 | **1.00** | 0.09 | 0.30 | 0.18 | 0.00 | 0.07 | 0.01 |
|  | *ph.ox.* | 0.03 | 0.36 | 0.32 | 0.13 | 0.14 | 0.07 | **1.00** | **0.66** | 0.11 | 0.01 | 0.32 | 0.36 |  | 0.35 | 0.31 | 0.10 | 0.30 | 0.15 | 0.12 | **1.00** | 0.40 | 0.04 | 0.03 | 0.00 | 0.01 |  | 0.12 | 0.34 | 0.20 | 0.13 | 0.14 | 0.09 | **1.00** | **0.52** | 0.01 | 0.00 | 0.02 | 0.11 |
|  | *perox* | 0.20 | **0.53** | 0.42 | 0.20 | 0.32 | 0.30 | **0.66** | **1.00** | 0.00 | 0.06 | 0.19 | 0.07 |  | 0.40 | 0.43 | 0.06 | 0.11 | 0.17 | 0.31 | 0.40 | **1.00** | 0.10 | 0.30 | 0.15 | 0.19 |  | 0.23 | 0.47 | 0.20 | 0.10 | 0.23 | 0.30 | **0.52** | **1.00** | 0.00 | 0.06 | 0.11 | 0.09 |
|  | *act prot* | 0.04 | 0.02 | 0.02 | 0.15 | 0.42 | 0.10 | 0.11 | 0.00 | **1.00** | 0.29 | 0.27 | **0.69** |  | 0.00 | 0.02 | 0.00 | 0.21 | 0.48 | 0.46 | 0.04 | 0.10 | **1.00** | 0.02 | 0.43 | 0.41 |  | 0.00 | 0.00 | 0.02 | 0.25 | 0.37 | 0.18 | 0.01 | 0.00 | **1.00** | 0.20 | 0.02 | 0.32 |
|  | *gluc p.* | 0.09 | 0.05 | 0.21 | 0.37 | 0.34 | 0.00 | 0.01 | 0.06 | 0.29 | 1.00 | 0.00 | 0.44 |  | 0.04 | 0.09 | 0.07 | 0.03 | 0.06 | 0.07 | 0.03 | 0.30 | 0.02 | **1.00** | 0.03 | 0.00 |  | 0.13 | 0.05 | 0.18 | 0.35 | 0.21 | 0.00 | 0.00 | 0.06 | 0.20 | 1.00 | 0.06 | 0.37 |

Presented are goodness of fit (*R*^2^). Statistical significant (*P* < 0.05) relationships are highlighted in grey: light grey, *R*^2^≤ 0.25; dark grey, *R*^2^>0.25.Bold values highlight *R*^2^ higher than 0.5. Regressions are based on data from control and girdling plots from all samplings over the 2-yr sampling period (mean values of each sampling). *Cellob*, cellobiosidase; *chitin*, Chitinase; *N-acet*, N-acetyl-glucosaminidase; *L-pept*, Leucine-peptidase; *ph.ox*., phenoloxidase; *perox*, peroxidase; *act prot*, actual protease; *gluc p*., glucose production; *dN*, total dissolved nitrogen; *bact*., bacteria; *fung*, fungi; *f&p*, fungi and plant.
